# Negative Impact of the UEFA European Soccer Championship on Central Hemodynamics and Arterial Stiffness: A Multicenter Study

**DOI:** 10.3390/life12111696

**Published:** 2022-10-25

**Authors:** Klaas F. Franzen, Kai Mortensen, Christian Ott, Katrin Herber, Marlene Busse, Charlotte Söling, Daniel Schneppe, Saskia Lässig, Marcus Dörr, Roland Tilz, Daniel Drömann, Heribert Schunkert, Michael Reppel

**Affiliations:** 1 Medical Clinic III, Campus Lübeck, University Hospital Schleswig-Holstein, 23562 Lubeck, Germany; 2 Airway Research Center North, Member of the German Center for Lung Research (DZL), 22927 Großhansdorf, Germany; 3 Cardiology Kiel, 24116 Kiel, Germany; 4 Clinic for Rhythmology, Campus Lübeck, University Hospital Schleswig-Holstein, 23562 Lubeck, Germany; 5 Department of Nephrology and Hypertension, Friedrich-Alexander University Erlangen-Nürnberg, 91054 Erlangen, Germany; 6 Medical Clinic B, University Hospital Greifswald, 17491 Greifswald, Germany; 7 Deutsches Herzzentrum München, Klinik für Herz- und Kreislauferkrankungen, Technische Universität München, 80333 Munich, Germany; 8 Deutsches Zentrum Für Herz- und Kreislauf-Forschung (DZHK) E.V. (German Center for Cardiovascular Research), Partner Site Munich Heart Alliance, 80333 Munich, Germany; 9 Technical University of Munich, 80333 Munich, Germany; 10 Cardiology Landsberg, 86899 Landsberg, Germany

**Keywords:** emotional stress, European soccer championship, arterial stiffness, central hemodynamics, cardiovascular disease

## Abstract

(1) Background: watching sporting events may trigger cardiovascular events by elevating emotional stress levels. The underlying reasons and specific populations at risk are not well defined. (2) Methods: we conducted a multicenter prospective trial at three German sites during the UEFA Soccer EC 2012 and 2021 comprising 52 healthy participants (noCVD) and 18 patients hospitalized with cardiovascular disease (CVD). Subjects were studied during matches of the German national team (GP) as well as corresponding matches without German participation (noGP). Peripheral and central blood pressure (BP) and parameters of arterial stiffness were measured (Mobil-O-Graph™, I.E.M., Stolberg, Germany) before, during, and after the matches. (3) Results: in terms of CVD, peripheral as well as central BP and heart rate increased significantly during GP as well as noGP matches and remained elevated beyond the end of the matches. Likewise, arterial stiffness parameters and vascular resistance were higher during the matches and remained elevated after the matches. No consistent significant differences were found between GP and noGP matches. (4) Conclusions: this is the first study on real-life changes in hemodynamics during sport-associated emotional stress, with comparison between noCVD and CVD. We found that alterations were profound in CVD and remained elevated even after the matches.

## 1. Introduction

Several reports have described the negative effect of emotional stress on cardiovascular events [1,2,3,4]. In addition to other triggers, watching soccer in major events may potentially increase cardiovascular risk [5,6].

One of the first trials on the soccer-associated increase in cardiovascular risk, conducted in 1996, showed the negative effects of the quarter-final match of the UEFA European Championship between the Netherlands and France in the form of increased cardiovascular events and mortality [7]. During the World Cup 1998 in France, the risk of admission to hospital for acute myocardial infarction was elevated by approximately 25% on 30 June 1998 in a British population when England lost the penalty shoot-out against Argentina and also in the following two days [8]. In line with these data, Wilbert-Lampen and coworkers studied a German population during the Soccer World Cup 2006 and reported a 2–3-fold increase in the incidence of acute cardiovascular events compared with the control period of a non-matchday of the German national team [9]. It was found that the first two hours after the beginning of the German team matches was associated with the highest risk and incidence of cardiovascular events, which was higher in men than in women (3.26- versus 1.82-fold increase) [6]. The number of deaths caused by myocardial infarction, however, was not elevated compared with the control period [6]. Borges et al. also found an impact of World Cup soccer games on the incidence of myocardial infarction without affecting in-hospital mortality [10].

Barone-Adesi et al. studied approximately 25,000 hospital admissions for acute myocardial infarction among the Italian population during the World Cup 2002, the UEFA European Championship 2004, and the World Cup 2006. They did not observe increased numbers of cardiovascular events on the match days involving the Italian team [11]. Likewise, Niederseer et al. did not find an increased incidence of cardiac events in a retrospective analysis [12], and Jauss and coworkers found no increase in cerebrovascular events during the World Cup 2006 [13].

The pathophysiological relationship between stressful situations and acute cardiac events has been addressed by various studies and, generally, using different approaches [1,8,11,12]. Studies from Munich have looked at the events or blood samples themselves, which leaves open the possibility of retrospective analyses of myocardial infarction cases [8]. Whether noninvasive measurements can help elucidate these effects is unclear. Endothelial dysfunction and increased central hemodynamic load, reflected by central pulse pressure, pulse wave velocity (PWV), and augmentation index (AIx75) as a read-out parameter of arterial stiffness, are also used to predict cardiovascular risk [14,15,16,17]. The parameters of arterial vascular stiffness are considered surrogate parameters for cardiovascular events; the elasticity of vessels thus indirectly reflects indications of endothelial dysfunction, i.e., a damaged endothelium. Arterial vascular stiffness can be reproduced as a parameter of biological vessel age. PWV describes the running speed of the arterial pulse wave from the heart to the periphery, and the augmentation index describes the summation of the ejected pressure wave and reflected pressure wave over the arterial vascular system [18,19]. Acute emotional stress leads to an increase in arterial stiffness [20]. Of importance, central blood pressure and arterial stiffness are shown to have higher performance in predicting CV risk compared with peripheral blood pressure [21,22,23,24].

In addition to this modulation of vascular function, acute stress was found to increase leucocyte migration to tissues in a cellular/tissue-based mouse model [25]. This migration into atherosclerotic plaques via norepinephrine-dependent modulation of endothelial cells resulted in inflammatory cell accumulation and is believed to further increase vascular instability in terms of vascular inflammation and subsequent atherosclerotic plaque progression. 

In summary, stress is possibly a crucial risk factor for disruptions in vascular bed and function, and it could thereby contribute to significant cardiovascular morbidity and mortality. It is unclear which individuals are most at risk. Therefore, various groups are of interest: patients who suffer from CVD, in which there may be effects on endothelial dysfunction and diseased vessels; and a healthy control group with a healthy endothelium and vessels as intact as possible. We therefore aimed to study the effects of watching soccer on peripheral and central hemodynamics and arterial stiffness parameters in healthy individuals and patients suffering from CVD during European UEFA Soccer Championships.

## 2. Materials and Methods

### 2.1. Study Cohort and Design

This multicenter study included 70 German soccer fans and was carried out at the university hospitals of Lübeck, Erlangen, and Greifswald. Inclusion criteria were age ≥ 18 years and written informed consent. Two different subgroups were studied: (1) healthy (*n* = 52, exclusion criteria: mental disorders, ECG abnormalities, diabetes, cardiovascular disease (CVD), pregnancy, hypertension, and Karnofsky Index of at least 100%); and (2) hospitalized patients (*n* = 18) with known CVD (exclusion criteria: mental disorders and pregnancy). Details of the patient characteristics are shown in Table 1. The ethics committee approved the study (AZ-12-043).

All measurements were carried out during the UEFA Soccer European Championships 2012 or 2021. According to the guidelines for measuring arterial stiffness [19,21], caffeine, alcohol, or smoking cigarettes were forbidden at least 24 h before the matches and until the end of recordings. All participants were asked to avoid large meals at least 4 h before the measurements. Measurements were started at least 90 min before kick-off. Measurements were repeated every 15 min before kick-off and every 5 min during the game and were stopped 90 min after the end of the games. Group matches as well as end round matches with German participation (GP) were compared with corresponding matches without German participation (noGP), as listed in Table 1. CVD patients were compared with healthy individuals. 

### 2.2. Measurement of Peripheral and Central Blood Pressure and Arterial Stiffness

Blood pressure and central hemodynamics, as well as arterial stiffness, were measured at the arteria brachialis with the validated oscillometric Mobil-O-Graph™ devices (I.E.M.) (I.E.M., Stollberg, Germany) [26,27,28]. Before and after the matches, a mean value was calculated at 30 min intervals based on two individual measurements. During matches, a mean value was calculated every 15 min from three measurements at 5 min intervals. Overtime was included in the second-half time. 

### 2.3. Statistical Analysis 

Statistical analysis was performed using SPSS statistical software (SPSS, Inc., v. 23, Chicago, IL, USA). Graphs were edited using GraphPad Prism 5.0 (GraphPad Software, Inc., San Diego, CA, USA) and CorelDraw 11.0 (Corel Inc., Mountain View, Santa Clara County, CA, USA). Baseline values were taken as a statistical reference. Results were tested for normality of distribution using Kolmogorov–Smirnov tests. Paired Student *t*-tests and Wilcoxon tests were used where applicable, e.g., for comparison of the same time points of GP and noGP matches. Analysis of variance (ANOVA) was used for different time points of measurements and comparison of groups. A multivariate analysis of variance (MANOVA) allowed for correcting for age, mean arterial pressure (MAP), heart rate (HR), and sex. Unless otherwise stated, all data are expressed as mean ± standard error (SEM). A *p*-value < 0.05 was considered to indicate a statistically significant difference.

## 3. Results

### 3.1. Baseline Characteristics

Baseline characteristics for the entire group (*n* = 70), as well as healthy volunteers and CVD patients separately, are presented in Table 2. Two patients were lost to follow-up before the second game and four healthy fans were excluded due to a repetitively elevated baseline systolic blood pressure above 140 mmHg before the matches. In total, data from 139 watched games and 7089 measurement time points were analyzed.

### 3.2. Peripheral Hemodynamics Are Increased by Watching Soccer Games—Effects in noCVD

**Figure 1:**
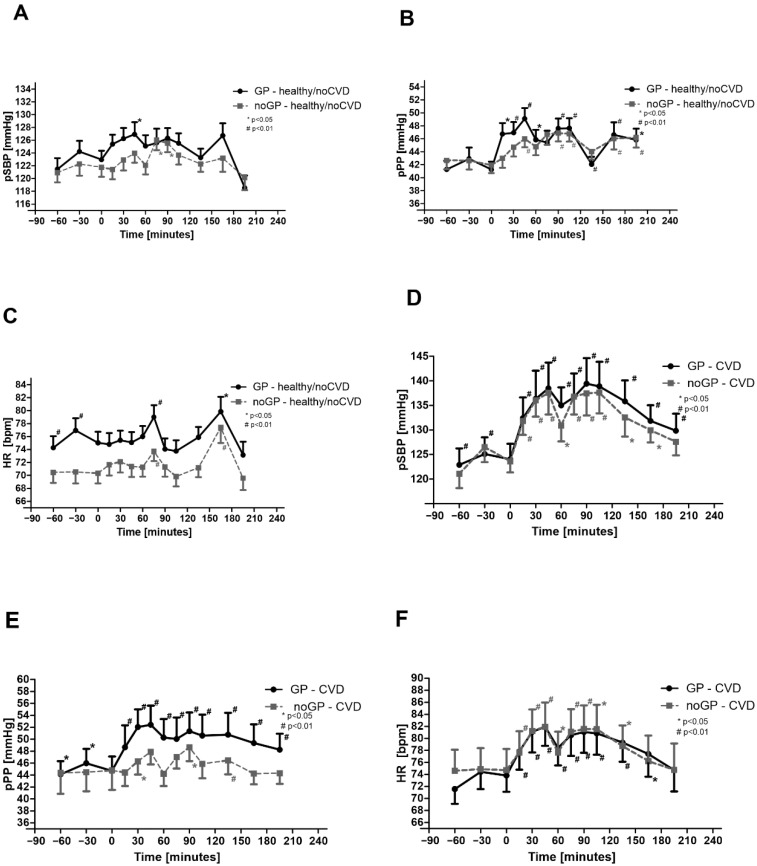
Peripheral blood pressure parameters: (**A**) peripheral systolic blood pressure (pSBP) in noCVD, (**B**) peripheral pulse pressure (pPP) in noCVD, (**C**) heart rate (HR) in noCVD, (**D**) peripheral systolic blood pressure (pSBP) in CVD, (**E**) peripheral pulse pressure (pPP) in CVD, and (**F**) heart rate (HR) in CVD. Asterisks (# means *p* < 0.01; * means *p* < 0.05) indicate a significant reduction in blood pressure values compared with baseline. Data are expressed as mean ± SEM.

In noCVD/noGP matches, peripheral systolic blood pressure (pSBP) showed a trend toward higher values throughout the matches; the differences became significant before the match end compared with the baseline values and returned almost to baseline after the matches. In noCVD/GP matches, pSBP values were consistently higher than for noCVD/noGP matches. However, differences between noGP and GP were not statistically significant. Although, pSBP values showed a significant interaction between time and group (Figure 1a). 

Effects were stronger for peripheral pulse pressure, with several significant group differences at different time points, which indicates a significant interaction between time and group (pPP, Figure 1b). For heart rate, we observed consistently higher values for noCVD/GP than for noCVD/noGP matches. Heart rate was also significantly elevated before the end of the matches and returned to baseline by the end of the matches for both GP and noGP matches (Figure 1c).

### 3.3. Peripheral Hemodynamics Are Increased by Watching Soccer Games—Effects in CVD

In CVD/noGP, we found strong effects on pSBP throughout matches and even after the end (Figure 1d). No significant differences were found between noGP and GP. The pPP was increased, especially in GP matches (Figure 1e), and the heart rate was significantly elevated in noGP and GP matches (Figure 1f).

### 3.4. Central Hemodynamics Are Increased by Watching Soccer Games—Effects in noCVD

**Figure 2:**
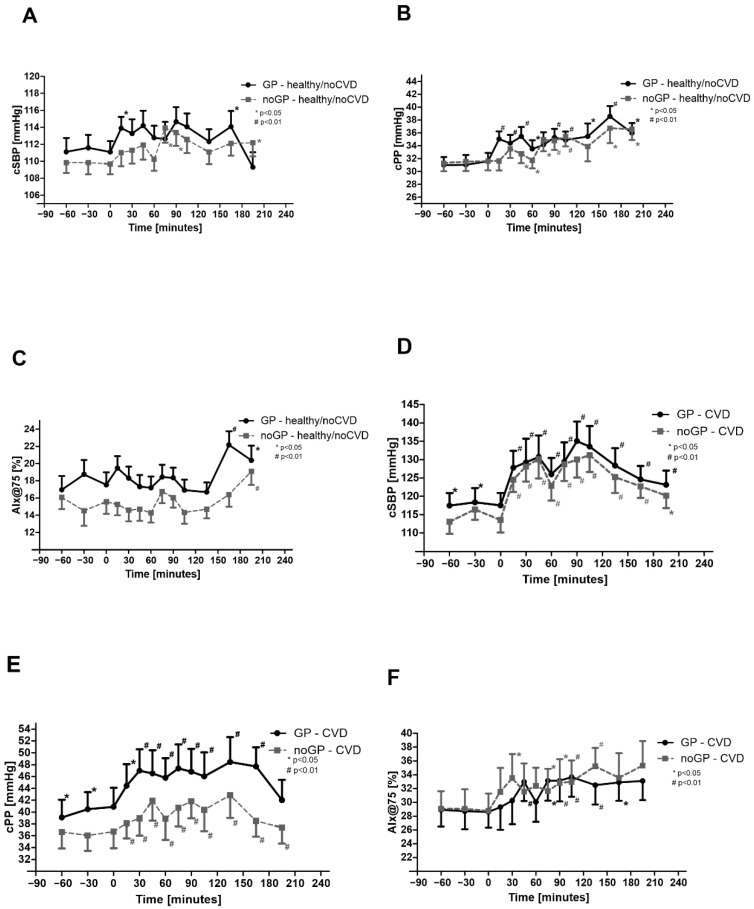
Central blood pressure parameters: (**A**) central systolic blood pressure (cSBP) in noCVD, (**B**) central pulse pressure (cPP) in noCVD, (**C**) augmentation index adjusted at 75 bpm (AIx@75) in noCVD, (**D**) central systolic blood pressure (cSBP) in CVD, (**E**) central pulse pressure (cPP) in CVD, and (**F**) augmentation index adjusted at 75 bpm (AIx@75) in CVD. Asterisks (# means *p* < 0.01; * means *p* < 0.05) indicate a significant reduction in blood pressure values compared with baseline. Data are expressed as mean ± SEM.

In noCVD, central cSBP and cPP values showed a similar pattern to the corresponding peripheral values (Figure 2a,b). AIx75 was not significantly affected throughout the matches but rose significantly after the end of the matches (Figure 2c). Although there were also found significant interaction between time and groups for AIx75 within noCVD.

### 3.5. Central Hemodynamics Are Increased by Watching Soccer Games—Effects in CVD

Significant elevations compared with baseline were observed for cSBP and cPP as well as AIx@75 in CVD (Figure 2d–f). For cPP, a significant difference was found between min 150 and 180 and GP/noGP matches (Figure 2e). As in noCVD, significant interactions were also found between time and groups for AIx75 within CVD.

### 3.6. Total Vascular Resistance and Pulse Wave Velocity in noCVD

**Figure 3:**
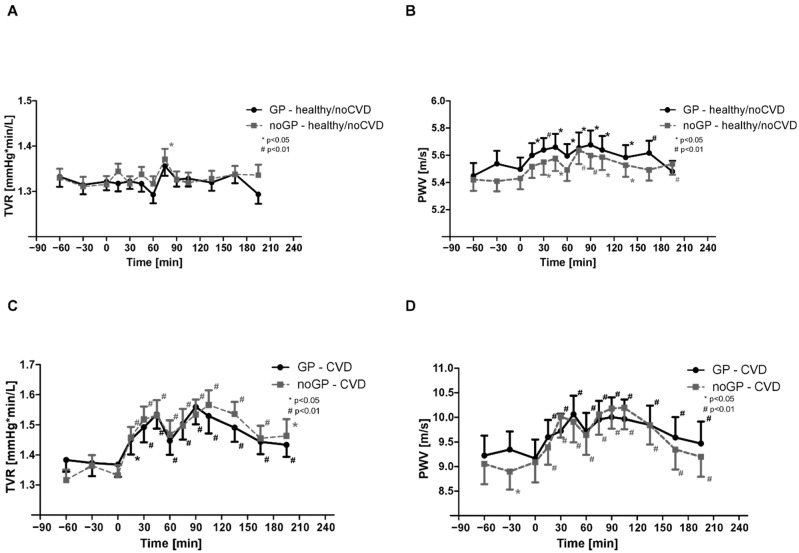
Total vascular resistance and pulse wave velocity: (**A**) total vascular resistance (TVR) in noCVD, (**B**) pulse wave velocity (PWV) in noCVD, (**C**) total vascular resistance (TVR) in CVD, and (**D**) pulse wave velocity (PWV) in CVD. Asterisks (# means *p* < 0.01; * means *p* < 0.05) indicate a significant reduction in individual values compared with baseline. Data are expressed as mean ± SEM.

Compared with the baseline, there was a significant increase in TVR between 60 and 90 min only in noGP and GP matches, respectively, but we did not find consistent changes during and after the matches (Figure 3a). PWV was significantly increased as early as 30 min after the start of the matches and remained elevated, even after the end of the matches, in both groups (Figure 3b). Further analysis showed also a significant interaction for time and group.

### 3.7. Total Vascular Resistance and Pulse Wave Velocity in CVD

Significant changes were observed for TVR and PWV throughout and after the matches in the CVD group, while there were no differences between noGP and GP matches (Figure 3c,d). Further analysis showed a significant interaction for time.

### 3.8. Comparison of noCVD and CVD

**Figure 4:**
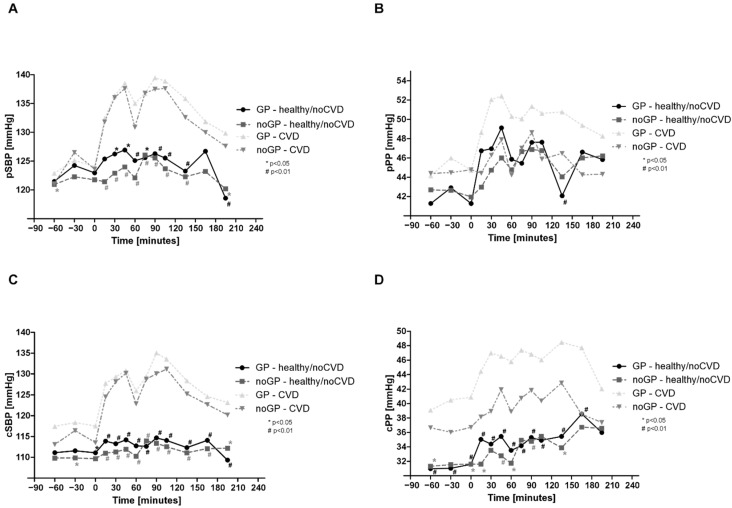
Comparison of peripheral and central hemodynamics in noCVD and CVD: (**A**) peripheral systolic blood pressure (pSBP), (**B**) peripheral pulse pressure (pPP), (**C**) central systolic blood pressure (cSBP), and (**D**) central pulse pressure (cPP). Asterisks (# means *p* < 0.01; * means *p* < 0.05) indicate a significant reduction in individual values compared with baseline. Data are expressed as mean.

In the comparison of noCVD and CVD, pSBP and pPP were similar at baseline. Values of pSBP, pPP, and cSBP while watching the matches were significantly higher in CVD than in noCVD (Figure 4a,c,d). No significant difference was found for pPP during the matches (Figure 4b).

**Figure 5:**
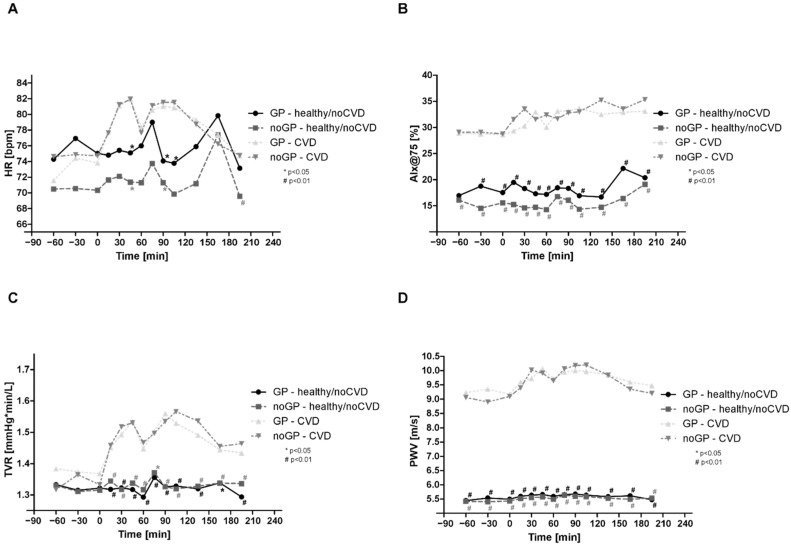
Comparison of heart rate and arterial stiffness in noCVD and CVD: (**A**) heart rate (HR), (**B**) augmentation index adjusted at 75 bpm (AIx@75), (**C**) total vascular resistance (TVR), and (**D**) pulse wave velocity (PWV). Asterisks (# means *p* < 0.01; * means *p* < 0.05) indicate a significant reduction in individual values compared with baseline. Data are expressed as mean.

Heart rate was higher in CVD and showed significantly higher values at 45 and 90 min, and between 90 and 120 min (Figure 5a).

Strong differences were observed for AIx@75 (Figure 5b), TVR (Figure 5c), and PWV (Figure 5d). There are not only significant differences between the two groups noCVD and CVD at baseline, but also significant differences between the individual time points compared to each baseline.

## 4. Discussion

In the present study, we show, for the first time, acute changes in peripheral and central blood pressures as well as in arterial stiffness parameters in healthy individuals and CVD patients during the emotional stress of watching a sporting event. The changes were much stronger in CVD patients than in healthy controls. Recent studies have described increased cardiovascular risk due to emotional stress [24,29,30]. However, until now, the underlying reasons and effects on central hemodynamics, as well as arterial stiffness, have remained unclear, especially in patients suffering from CVD while watching sporting events. In a previous study during a FIFA World Cup, we observed increased central pressure and arterial stiffness in a young and healthy population [31]. Similar changes were observed, though not as profound, during the UEFA European Championships 2012 and 2021. The qualifying round, which was possibly less thrilling during a UEFA European Championship than during a FIFA World Championship, and the early dropout of the competition during the European Championship in 2021 are possible explanations. The German team only reached the round of 16 in 2021, whereas they reached the semifinal during the World Cup and had a third-place match in 2010. These differences might also explain why otherwise similar studies from different nations could not find significant differences in risk [11].

Most interestingly, the changes in hemodynamic parameters were significantly more pronounced in CVD patients than in healthy individuals. Although CVD patients were older than the healthy subgroup, the PWV was at similar levels to healthy individuals of a similar age [32]. Thus, either the higher age or the cardiovascular disease itself might explain our findings. Although CVD patients were treated with cardioactive drugs, such as betablockers, heart rate was higher throughout the matches. Thus, we assume higher sympathetic nervous system activity in CVD than in healthy individuals, leading to increased vasoconstriction, as has been shown earlier [33,34,35].

In addition, as has been shown by Wilbert-Lampen and coworkers, increased sympathetic activity and vascular/endothelial mediators may have mediated prolonged increased vascular resistance/endothelial dysfunction, even after the end of the matches [5]. They showed that higher rates of stress-related CV events were mediated by increasing vasoconstrictive mediators, such as sCD40L, sVCAM-1, MCP-1, TNF-alpha, and ET-1. In line with this hypothesis, cardiovascular events are highest during the first 2 h after the beginning of World Cup soccer matches, i.e., even after the end of matches [6].

Due to the stronger effects on hemodynamics, CVD patients seem to be at higher risk in the context of emotional stress of watching a sporting event. A recent study by Hinterdobler et al. demonstrated stress-induced and norepinephrine/macrophage-mediated enhancement of leucocyte recruitment to atherosclerotic plaques [30,36]. As a consequence, inflammatory plaque leucocytes could increase vascular inflammation and plaque rupture.

Peripheral vasoconstriction is the result of stress, whether emotionally or physically mediated. Differences in the physiological effects of short-term versus long-term stress and the different forms of stress (‘positive’ versus ‘negative’) are still not entirely clear. In the context of emotional stress when watching sporting events, positive stress would be triggered by the victory of one’s own team and negative stress by a defeat. Short-term stress and physiological concentrations of stress hormones are believed to be immuno-enhancing, whereas chronic stress, with higher concentrations of stress hormones than physiological stress, may have dysregulatory effects [30,37]. It is well accepted that ‘negative’ stress, such as war [38], terrorism, or crime, leads to negative effects and increased cardiovascular risk [39], such as takotsubo cardiomyopathy [40] and myocardial infarction [11,41,42]. By contrast, the findings in the Munich cohort [11] hint at a stress-mediated negative outcome even if the German national team won the respective matches (‘positive stress’) [43]. Most likely, ‘positive stress’ would also increase the CV risk via the abovementioned immunomodulatory response and the sympathetic nervous system-mediated cascade, i.e., increased total peripheral resistance by vasoconstrictive mediators, peripheral and central pressure, as well as arterial stiffness parameters such as PWV and AIx@75 in at-risk populations.

Taken together, stress per se leads to hemodynamic effects that may potentially be harmful, especially in patients with CVD. The elevated sympathetic nervous system activity and preexisting vascular damage predispose these patients to negative outcomes during emotional stress. Thus, both ‘positive’ and ‘negative’ emotional stress can lead to the same negative effects on the vascular system.

In terms of the limitations of the present study, the number of noCVD and CVD individuals was limited, although measurements were extended to two UEFA Soccer Championships. The study was further limited (i) because of the complexity of the experimental setup, i.e., all individuals had to be measured during a noGP and GP match, (ii) by the low number of matches, and (iii) by the early dropout of the German team during the UEFA Championships. We cannot rule out that the higher age of the CVD patients influenced our findings, even though PWV was at similar levels in a healthy cohort [31]. Although all participants were selected by fan status, it is unknown whether noCVD individuals had the same enthusiasm as the CVD patients. Nevertheless, even if the noCVD group was less enthusiastic, the effects would most likely be more pronounced in more highly excited healthy individuals. Moreover, stress hormone levels are unknown. Future studies should address the endothelial mediators of our findings and determine the direct correlation between emotional stress levels and specific vascular effects. In addition, the control group is a further limitation when both groups were compared with each other. Basically, it was a healthy control group, but the members were significantly younger compared to the cohort with the cardiovascular patients, so an age-matched group would be more appropriate.

## 5. Conclusions

In conclusion, sporting events in general, because they are often associated with short-term emotional stress, might involve potential increased risk for CVD patients. Because stress levels may fluctuate highly when watching soccer, for example, depending on the team’s tactic (offensive/defensive), comparison with other, faster sports with a more constant high emotional stress level, such as ice hockey or basketball, would help us to determine the correlations between watching sports, stress levels, hemodynamics, and potential CV risk. Although hampered by interindividual differences, future studies should work on a gradation of ‘positive’ and ‘negative’ stress levels and link them to positive or negative effects on hemodynamics and immune system/vascular function.

## Figures and Tables

**Table 1:** Gameplan with matches with German participation and without German participation during the UEFA European Soccer Championships 2012 and 2021.

**UEFA European Soccer Championship 2012—Poland/Ukraine**		
	**noGP**	**GP**
**Group stage**	Ireland/Croatia	Germany/Portugal
	(1:3, 10 June 2012, 8:45 p.m.)	(1:0, 9 June 2012, 8:45 p.m.)
	Spain/Ireland	The Netherlands/Germany
	(4:0, 14 June 2012, 8:45 p.m.)	(1:2, 13 June 2012, 8:45 p.m.)
	Czech Republic/Poland	Denmark/Germany
	(1:0, 16 June 2012, 8:45 p.m.)	(1:2, 17 June 2012, 8:45 p.m.)
**Quarterfinals**	Czech Republic/Portugal	Germany/Greece
	(0:1, 21 June 2012, 8:45 p.m.)	(4:2, 22 June 2012, 8:45 p.m.)
**Semifinals**	Portugal/Spain	Germany/Italy
	(2:4, 27 June 2012, 8:45 p.m.)	(1:2, 28 June 2012, 8:45 p.m.)
**UEFA European Soccer Championship 2021—Europe**		
	**noGP**	**GP**
**Group stage**	Spain/Sweden	France/Germany
	(0:0, 14 June 2012, 9:00 p.m.)	(1:0, 15 June 2012, 9:00 p.m.)
	Croatia/Czech Republic	Portugal/Germany
	(1:1, 18 June 2012, 6:00 p.m.)	(2:4, 19 June 2012, 6:00 p.m.)
	Czech Republic/England	Germany/Hungary
	(0:1, 22 June 2012, 9:00 p.m.)	(2:2, 23 June 2012, 9:00 p.m.)
**Round of 16**	Croatia/Spain	England/Germany
	(3:5, 28 June 2012, 6:00 p.m.)	(2:0, 29 June 2012, 6:00 p.m.)

**Table 2:** Baseline characteristics.

Baseline Characteristics			
	All (*n* = 70)	Healthy/noCVD (*n* = 52)	CVD (*n* = 18)
Age (years)	37 ± 11	29 ± 8	54 ± 9
Sex	35 male, 35 female	23 male; 29 female	12 male; 6 female
Height (m)	1.54 ± 6.5	154.9 ± 7.4	1.50 ± 13.6
Weight (kg)	73.4 ± 1.6	70.2 ± 1.7	81.6 ± 3.4
Smoking (%)	11.6	0	42.1
Arterial hypertension (%)	24.6	0	84.2
Diabetes mellitus Type 1 and 2 (%)	8.7	0	26.3
ACS (%)	11.6	0	42.1
STEMI (%)	8.7	0	31.6
NSTEMI (%)	7.2	0	26.3
Coronary heart disease (%)	13.0	0	47.4
Heart failure—NYHA 1–2 (%)	8.7	0	31.6
Heart failure—NYHA 3 (%)	2.9	0	10.5
Atrial fibrillation (%)	1.4	0	5.3
Mitral valve insufficiency (%)	1.4	0	5.3
Tricuspidal valve insufficiency (%)	1.4	0	5.3
ICD (%)	5.8	0	21.1
Chronic kidney disease (%)	4.3	0	15.8
Hyperlipoproteinemia (%)	8.7	0	31.6
Arterial occlusive disease (%)	1.4	0	5.3
Bronchial asthma (%)	1.4	2	0
Beta blocker (%)	15.9	0	57.9
Aspirin (%)	11.6	0	36.8
Spironolactone (%)	7.2	0	26.3
ACE inhibitors (%)	14.5	0	52.6
ARB (%)	2.9	0	10.5
Statins (%)	20.3	0	73.7
Ticagrelor (%)	1.4	0	5.3
Clopidogrel (%)	5.8	0	21.1

## Data Availability

Not applicable.
